# Persistent sciatica induced by quadratus femoris muscle tear and treated by surgical decompression: a case report

**DOI:** 10.1186/1752-1947-4-236

**Published:** 2010-08-02

**Authors:** Artan Bano, Apostolos Karantanas, Dritan Pasku, George Datseris, George Tzanakakis, Pavlos Katonis

**Affiliations:** 1 Department of Orthopaedic and Traumatology, University Hospital of Heraklion, 71110, Crete, Greece; 2 Department of Radiology, University Hospital of Heraklion, 71110, Crete, Greece; 3 Department of Pathology, Medical School, University of Crete, Heraklion, 71003, Greece; 4 Department of Histology, Medical School, University of Crete, Heraklion, 71003, Greece

## Abstract

**Introduction:**

Quadratus femoris tear is an uncommon injury, which is only rarely reported in the literature. In the majority of cases the correct diagnosis is delayed due to non-specific symptoms and signs. A magnetic resonance imaging scan is crucial in the differential diagnosis since injuries to contiguous soft tissues may present with similar symptoms. Presentation with sciatica is not reported in the few cases existing in the English literature and the reported treatment has always been conservative.

**Case presentation:**

We report here on a case of quadratus femoris tear in a 22-year-old Greek woman who presented with persistent sciatica. She was unresponsive to conservative measures and so was treated with surgical decompression.

**Conclusion:**

The correct diagnosis of quadratus muscle tear is a challenge for physicians. The treatment is usually conservative, but in cases of persistent sciatica surgical decompression is an alternative option.

## Introduction

Traumatic quadratus femoris muscle tear is a clinically unsuspected injury. The immediate and correct diagnosis is a challenge because of its rarity and similarities to other disorders that cause groin pain. Only a few cases of partial and complete rupture of quadratus femoris muscle in the young active population have been reported in the literature [1,2]. In all cases, magnetic resonance imaging (MRI) was crucial both in correct diagnosis and guidance of treatment. Simultaneously, different therapeutic techniques were used including the injection of methylprednisolone acetate (Depo-Medrol), transcutaneous neurostimulation, ultrasound and physical rehabilitation techniques [1]. We present a rare case of quadratus femoris muscle rupture associated with persistent sciatica, which was treated with surgical decompression.

## Case presentation

**Figure 1:**
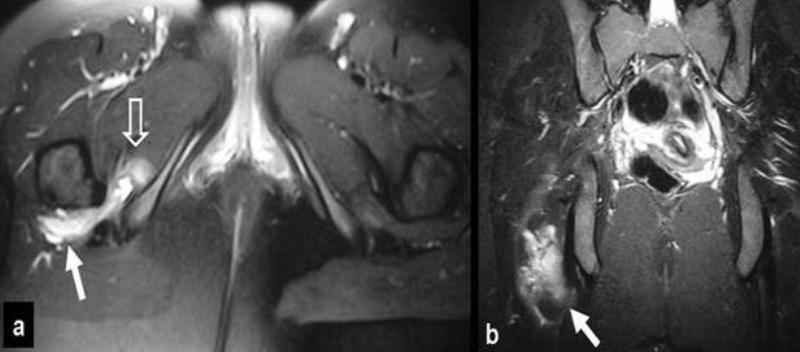
**MRI performed a few days after injury**. (a) The transverse fat suppressed proton density turbo spin echo (TSE) and (b) the coronal short tau inversion recovery (STIR) images, show the hematoma formation in the quadratus femoris muscle (arrows) extending to the obturator internus muscle (open arrows).

A 22-year-old Greek woman sustained a direct injury to the right buttock following a fall down the stairs. After the injury she had an antalgic gait due to pain in the right inferior gluteal area with radiation to the proximal posterior thigh. Pain was aggravated by sitting and squatting. MRI examination at that time revealed an extensive hematoma extending to both the quadratus femoris and obturator externus muscles, in keeping with strain grade II (Figure 1). She was treated with non-steroidal anti-inflammatory drugs (NSAID) without improvement.

**Figure 2:**
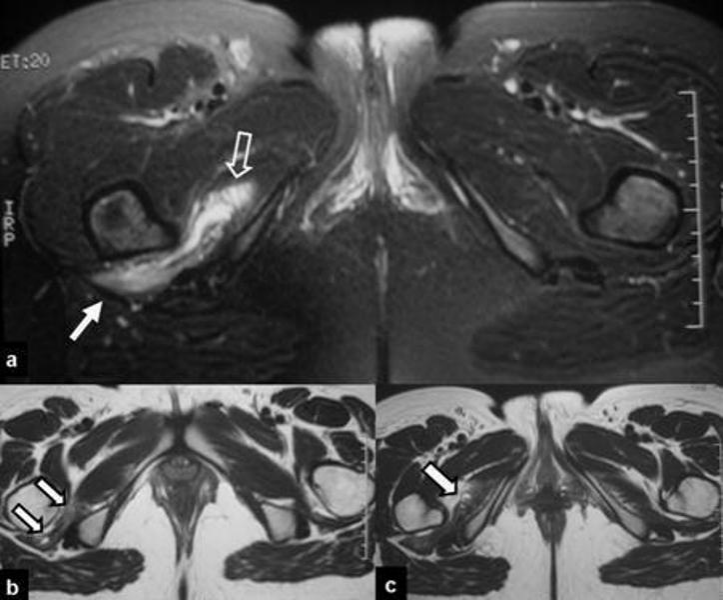
**The follow-up MRI examination was performed six months later**. (a) The transverse fat suppressed proton density (PD)-weighted TSE image, shows persistent dimensions of the hematoma-like lesion in the quadratus femoris (arrow) and the obturator internus (open arrow) muscles. The corresponding T1-weighted spin echo (SE) images show the high signal intensity on the bone-tendinous junction of the quadratus femoris (arrows in b) and obturator internus (arrow in c). These areas histologically turned out to correspond to a mixture of chronic hematoma, fibrosis, granulation tissue and fatty infiltration.

**Figure 3:**
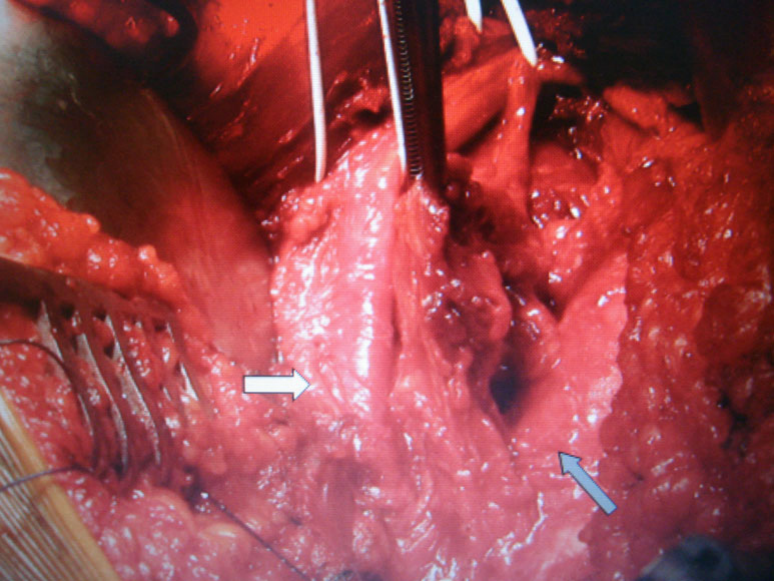
**Intra-operative picture showing the sciatic nerve (white arrow) and the ruptured quadratus muscle (black arrow)**.

**Figure 4:**
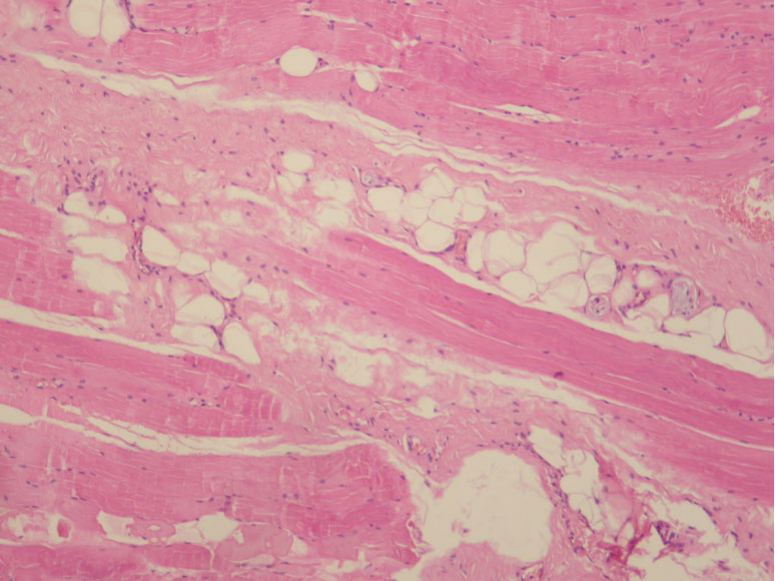
**Hematoxylin and eosin stain, magnification ×400 (×400, H&E)**. Histopathological examination of the removed mass showing a significant quantity of fibrotic tissue and atrophy of muscles bundles.

Six months after the injury, she was referred to our tertiary health care hospital for consultation due to persistent sciatica. Physical examination revealed an active young woman with healthy muscular development. There were no abnormalities on examination, such as soft tissue swelling, ecchymosis or erythema of the right gluteus and lower leg. There was tenderness upon palpation at the right ischial tuberosity associated with reduced muscular strength at right hip external rotators. Right straight leg rising (SLR) reproduced symptoms at 30° and her Lasegue test was positive. Passive hip internal rotation also reproduced pain in the proximal posterior thigh, with positive Freiberg and flexion, adduction, internal rotation (FADIR) tests. Her vascular clinical tests and the lumbar spine examination were normal. Standard hip, lumbar spine and pelvis radiographs were unremarkable. The complete laboratory work-up did not reveal any indication for infection or coagulopathy. Based on the above, the initial clinical impression was piriformis syndrome. A new MRI examination of our patient was requested for confirmation. This showed that the previous muscular strain showed only a minor degree of hematoma absorption compared to the previous study (Figure 2). Thus, hematoma formation was thought to be responsible for her persistent sciatica. A conservative approach, by means of strengthening of our patient's external rotators muscles, did not show any improvement for one month. The lack of obvious fluid effusion did not allow computed tomography (CT)-guided drainage. A surgical exploration of our patient was then performed through a posterolateral approach of the right hip. Intra-operatively, an atrophic quadratus femoris muscle was found, with complete detachment at the tendon-bone junction from the quadrate tubercle of the femur (grade III strain) (Figure 3). An associated solid mass (5 cm × 2 cm × 3 cm), representing chronic hematoma and fibrosis, was attached to and compressed the sciatic nerve. After decompressing the sciatic nerve from the fibrotic and granulation tissue, the newly formed mass was evacuated. The greater trochanteric bursa and contiguous structures were noted to be normal. The histological findings were compatible with degenerative muscular changes including fibrotic tissue, significant atrophy and fatty replacement (Figure 4).

Post-operatively, management consisted of physical rehabilitation with emphasis on the strengthening of the external rotator muscles with pain-free isometric progressive exercises. One month after surgery, our patient was free of symptoms and returned to work. At the one-year follow-up, she had no abnormal symptoms or signs.

## Discussion

Post-traumatic pain located in the buttock area may develop following a pelvic or coccygeal fracture or a muscle strain, with hematoma resulting in sciatic nerve compression. A traumatic lumbar disc herniation may be found in patients with radicular pain. For a correct clinical evaluation it is essential to assess the osseous structures and the muscles around the hip joint. The quadratus femoris muscle is a flat quadrilateral muscle that arises from the upper external border of the ischial tuberosity and inserts at the quadrate tubercle of the femur [3]. It acts as a hip external rotator and assists adduction [3,4]. The quadratus femoris muscle is innervated by the quadratus femoris nerve which rises from the ventral roots of the L4, L5 and S1 nerves in 79.4% of patients [5]. In adults, the myotendinous junction is the most vulnerable location for injury [6,7]. The tendon insertion in the bone may also be affected.

Only a few cases describing a quadratus femoris muscle injury have been reported in the literature. The incidence of the quadratus femoris muscle tear is unknown. O'Brien and Bui-Mansfield presented a review of seven cases [1]. In this study, this type of injury occurs predominantly in women (as in our case) with a female to male ratio of 6:1. The age of patients ranges from 17 to 43 years with an average age of 29.6 years. The symptoms were hip pain in three patients, groin pain in one patient and deep posterior thigh or gluteal pain in three patients. In none of the cases reported was there a correct clinical diagnosis of quadratus femoris muscle tear. Diagnosis was confused with a hamstring injury, snapping hip syndrome or lumbar radiculopathy. The delay from time of injury to correct diagnosis varied from one day to five months [1]. In one case the injury was located at the tendon insertion and in the rest at the musculotendinous part. All cases were evaluated by MRI examination.

The exact mechanism of this injury is unknown. In tennis players it may result from a strong eccentric stress upon the quadratus femoris muscle in an attempt to control hip internal rotation during the follow-through phase of serving [2]. On the other hand, a congenitally smaller distance between the lesser trochanter and the ischial tuberosity is a predisposing factor for impingement of the quadratus femoris muscle [8].

The treatment of muscular strain consists of a carefully planned physical rehabilitation programme. In the first days after injury, progressive flexibility and pain-free strengthening exercises for the external hip rotator muscle should be performed. Then, the strengthening exercises are progressed to eccentric loading, as symptoms subside [2].

MRI has an important role in confirming the clinical suspicion, ruling out other soft tissue injuries and aiding prognosis [1,9,10]. Published case reports have shown the correlation of quadratus femoris tendinitis with groin pain [11] and muscle tear with hip pain [1]. According to O'Brien and Bui-Mansfield, axial T2-weighted fat-suppressed magnetic resonance (MR) images have demonstrated the presence of edema between the lesser trochanter and ischial tuberosity. On sagittal T2-weighted fat-suppressed images the edema is localized posterior to the lesser trochanter [1].

We suggest that in our case the grade III, quadratus femoris strain at the tendon-bone junction resulted in an organized mass which compressed the sciatic nerve, simulating piriformis syndrome. To our knowledge, this is the first case of quadratus femoris tear treated by open surgical decompression due to persistent sciatica.

## Conclusions

The primary symptoms of a severe quadratus femoris strain are buttock pain with posterior thigh pain, which is aggravated by sitting or activity, and reproduction of buttock pain on prolonged hip flexion, adduction and internal rotation. MRI is crucial in identifying this unusual injury and in excluding damage to neighbouring structures. However, due to the presence of extensive hematoma, imaging may downstage the degree of strain. The above injury should be considered in the differential diagnosis of any patient presenting with proximal thigh pain after injury. The therapy is usually conservative consisting of rehabilitation but, in the case of persisting symptoms, open sciatic nerve decompression should be an alternative approach.

## Consent

Written informed consent was obtained from the patient for publication of this case report and any accompanying images. A copy of the written consent is available for review by the Editor-in-Chief of this journal.

## Competing interests

The authors declare that they have no competing interests.

## Authors' contributions

DP, AB and PV initiated and co-wrote the paper and performed the surgical treatment of the muscle rupture. AK analyzed the MR images, prepared the illustrations, and performed the proof editing. GD and GT examined the specimen and prepared the histological illustrations of the excised mass. All authors have read and approved the final manuscript.
